# Effects of Metformin on Survival and Toxicity in Patients with Metastatic Non-Small Cell Lung Cancer Treated with Nivolumab

**DOI:** 10.3390/medicina61071161

**Published:** 2025-06-26

**Authors:** Heves Surmeli, Sedat Yildirim, Deniz Isik, Oguzcan Kinikoglu, Yunus Emre Altintas, Ugur Ozkerim, Sıla Oksuz

**Affiliations:** Department of Medical Oncology, Kartal Dr. Lütfi Kirdar City Hospital, Health Science University, 34865 İstanbul, Türkiye; rezansedat@hotmail.com (S.Y.); dnz.1984@yahoo.com (D.I.); ogokinikoglu@yahoo.com (O.K.); yunusaltıntas1688@gmail.com (Y.E.A.); ugur.ozkerim@hotmail.com (U.O.); sila.oksuz@gmail.com (S.O.)

**Keywords:** nivolumab, metformin, overall survival, thrombocytopenia, non-small cell lung cancer, demographics

## Abstract

*Background and Objectives:* This study evaluated the effects of concurrent metformin use on clinical outcomes in patients with metastatic non-small cell lung cancer (NSCLC) treated with nivolumab. *Materials and Methods:* A total of 152 patients were analyzed, including 110 non-users and 42 metformin users with type 2 diabetes. *Results:* A significant gender imbalance was observed, with a higher proportion of females in the metformin group (33.3% vs. 8.2%, *p* < 0.05). The metformin group showed a numerically higher body mass index (BMI), though not statistically significant (26.53 vs. 24.97, *p* = 0.065). Overall survival was significantly longer in the metformin group (5.02 ± 3.93 vs. 4.6 ± 3.79 years, *p* < 0.05), while progression-free survival did not differ significantly (1.32 ± 0.97 vs. 1.04 ± 0.75 years, *p* = 0.385). Although most adverse events were similar across groups, grade 3–4 thrombocytopenia was more frequent in metformin users (*p* < 0.05). Multivariate analysis showed that increased nivolumab treatment cycles were significantly associated with reduced mortality risk (OR = 0.64, 95% CI: 0.54–0.75, *p* < 0.05). *Conclusions:* These findings suggest that concurrent metformin use may enhance overall survival but also increase hematologic toxicity, warranting closer monitoring in NSCLC patients receiving nivolumab.

## 1. Introduction

### 1.1. Background to the Study

Nivolumab is a programmed death-1 (PD-1) immune checkpoint inhibitor that enhances T-cell-mediated immune responses against tumors. Landmark trials such as CheckMate 017 and 057 demonstrated that nivolumab significantly improved overall survival compared to docetaxel in previously treated NSCLC, with median OS of 9.2 months versus 6.0 months (CheckMate 017) and 12.2 months versus 9.4 months (CheckMate 057). These trials established nivolumab as a standard treatment in advanced NSCLC by prolonging overall survival (OS) compared to chemotherapy. 

Metformin, a first-line treatment for type 2 diabetes, has gained interest for its potential anticancer properties [1]. It is typically administered at doses ranging from 500 to 2000 mg/day. Emerging evidence suggests that metformin may influence tumor metabolism, reduce systemic inflammation, and modulate the immune microenvironment [2]. For instance, references [3,4,5] described mechanisms including activation of AMP-activated protein kinase (AMPK), reduction in myeloid-derived suppressor cells (MDSCs), and enhancement of cytotoxic T-cell responses. These mechanisms offer a potential rationale for combining metformin with immune checkpoint inhibitors like nivolumab.

Several preclinical and early clinical studies have investigated the synergy between metformin and immunotherapy. In models of lung and colorectal cancer, this combination has been shown to enhance antitumor immunity, although results remain variable across tumor types and clinical settings [6]. The low cost, well-established safety profile, and immunometabolic activity of metformin make it an attractive adjunct in cancer therapy, particularly in NSCLC, where durable responses are often limited to a subset of patients. However, rigorous, robust real-world data are scarce, especially regarding their effect on survival outcomes and immune-related adverse events in NSCLC patients receiving nivolumab [7].

This study aims to address that gap by evaluating survival, toxicity, and demographic outcomes among NSCLC patients treated with nivolumab with or without concurrent metformin use.

### 1.2. Research Gap

Despite the widespread clinical use of immune checkpoint inhibitors like nivolumab, the impact of concurrent metformin use on treatment outcomes remains unclear, particularly in patients with non-small cell lung cancer (NSCLC). Several retrospective studies, such as those by [8,9], suggest potential survival benefits when metformin is combined with immunotherapy. However, these studies often include heterogeneous cancer types and focus primarily on progression-free survival, with limited data on overall survival, immune-related toxicities, or demographic influences like sex and BMI. 

Moreover, existing literature frequently omits NSCLC-specific subgroup analyses and lacks adjustments for critical confounders such as diabetes status and gender distribution. This limits the generalizability and applicability of the findings in clinical practice. There is also little insight into whether metformin modifies the toxicity profile of nivolumab, particularly hematologic toxicities. Thus, there is a critical need for focused, real-world data evaluating the survival and toxicity outcomes of concurrent metformin use in NSCLC patients receiving nivolumab.

### 1.3. Objectives

This study aims to evaluate the effects of concurrent metformin use on survival outcomes, laboratory parameters, and treatment-related toxicities in patients with metastatic NSCLC treated with nivolumab. The primary objective is to compare overall survival (OS) and progression-free survival (PFS) between metformin users and non-users. Secondary objectives include assessing the incidence of adverse events—particularly thrombocytopenia—and analyzing whether demographic or clinical variables such as gender, BMI, and diabetes status influence these outcomes. By focusing on a real-world NSCLC cohort, this study seeks to clarify the potential role of metformin as an adjunct to immune checkpoint inhibition and explore whether specific patient subgroups derive greater benefit or experience increased risks.

## 2. Materials and Methods

### 2.1. Study Design

The study protocol was approved by the institutional review board of Kartal Dr. Lütfi Kirdar City Hospital (Decision No: 2025/010.99/11/6, dated 26 March 2025).

This retrospective observational study was conducted at a tertiary oncology center in Istanbul, Türkiye.

This retrospective cohort study analyzed data from 152 patients with metastatic non-small cell lung cancer (NSCLC) who received nivolumab therapy at a single tertiary oncology center between 2018 and 2023. The primary objective was to assess whether concurrent metformin use impacted survival and toxicity outcomes. Patients were stratified into two groups: 42 patients who received metformin for type 2 diabetes during nivolumab treatment and 110 patients who did not receive metformin. The study was designed to reflect real-world clinical settings, and metformin use was not randomized but based on pre-existing diabetes treatment.

### 2.2. Patient Selection

Eligible participants were adults (≥18 years) with histologically confirmed metastatic NSCLC treated with nivolumab. Patients were required to have complete medical records, including baseline demographics, laboratory data, treatment details, and follow-up outcomes. Exclusion criteria included receipt of other concurrent glucose-lowering medications, other active malignancies, or incomplete survival/toxicity data. Metformin users were defined as patients with type 2 diabetes who were prescribed oral metformin at 500–2000 mg/day during immunotherapy. Non-users included patients without diabetes or those not treated with glucose-lowering agents. A power analysis using G*Power 3.1.9.4 indicated that a sample size of 152 would provide 99.2% power to detect an effect size of 0.8 at α = 0.05.

### 2.3. Data Collection

Data were extracted from electronic health records and independently verified by two researchers. Collected variables included age, sex, body mass index (BMI), cancer histology, laboratory values (e.g., lymphocytes, neutrophils, platelets, monocytes), and treatment duration. Clinical endpoints were overall survival (OS), defined as time from nivolumab initiation to death or last follow-up; and progression-free survival (PFS), defined according to RECIST 1.1 criteria. Adverse events were graded using Common Terminology Criteria for Adverse Events (CTCAE) version 5.0, with specific attention to hematologic toxicities such as thrombocytopenia.

### 2.4. Statistical Analysis

Statistical analyses were performed using IBM SPSS Statistics version 26.0 (Armonk, NY, USA). Descriptive statistics included frequencies, percentages, means ± standard deviations, and medians with interquartile ranges. Normality was assessed using the Shapiro–Wilk test. Between-group comparisons were conducted using independent samples *t*-tests for normally distributed variables and Mann–Whitney U tests for non-normal distributions. Categorical variables were compared using chi-square or Fisher’s exact tests. Propensity score matching was conducted using R software (MatchIt version 4.5.2). Multicollinearity was checked using variance inflation factors. Model fit was assessed using Nagelkerke R^2^. A *p*-value < 0.05 was considered statistically significant.

To reduce selection bias and control for baseline differences between metformin users and non-users, a 1:1 propensity score matching (PSM) was performed using the nearest-neighbor method without replacement. Propensity scores were estimated using a logistic regression model that included the following covariates: age, sex, body mass index (BMI), cancer histology, and number of nivolumab treatment cycles.

A caliper width of 0.2 standard deviations of the logit of the propensity score was used. Balance between groups was assessed using standardized mean differences (SMDs), with SMD < 0.1 considered acceptable. Matched cohorts were then used for comparison of survival outcomes (OS and PFS) and adverse events.

Although laboratory parameters and additional clinical features (e.g., platelet count, lymphocyte levels) were not included in the propensity model due to sample size constraints, key demographic and treatment-related variables were balanced post-matching (SMD ≤ 0.1).

Correlations between variables were assessed using Spearman’s rank correlation. Multivariate logistic regression was performed to evaluate predictors of mortality, adjusting for age, sex, BMI, cancer histology, metformin use, and number of nivolumab cycles. OS and PFS were analyzed using Kaplan–Meier survival curves and compared using the log-rank test. A *p*-value < 0.05 was considered statistically significant. Missing data were excluded from the relevant analyses but not imputed

## 3. Results

### 3.1. Demographic and Laboratory Findings

A total of 14/42 (33.3%) of metformin users were female, compared to 9/110 (8.2%) non-users. A statistically significant difference was observed in gender distribution, with 33.3% of women in the metformin group compared to 8.2% in the non-user group (*p* < 0.05). Although a higher proportion of metformin users were aged ≥65 years (57.1% vs. 38.2%), the difference was not statistically significant (*p* = 0.054). The mean body mass index (BMI) was higher in metformin users (26.53 ± 4.54) than in non-users (24.97 ± 4.72), though the difference was not statistically significant (*p* = 0.065). Table 1 summarises the Demographic and laboratory comparisons in gender distribution.

Laboratory parameters, including lymphocytes, neutrophils, platelets, and monocytes, did not differ significantly between the two groups. Mean platelet counts appeared inconsistent due to a likely data entry error (reported as 295,704.3 vs. 275,563.64). These values should be verified for unit accuracy and biological plausibility. Post-matching, standardized mean differences for age, sex, BMI, histology, and nivolumab cycles were all ≤0.06, indicating good balance across groups. However, laboratory markers such as lymphocyte and monocyte counts were not matched and may still reflect residual imbalance.

### 3.2. Survival and Mortality Outcomes

**Figure 1:**
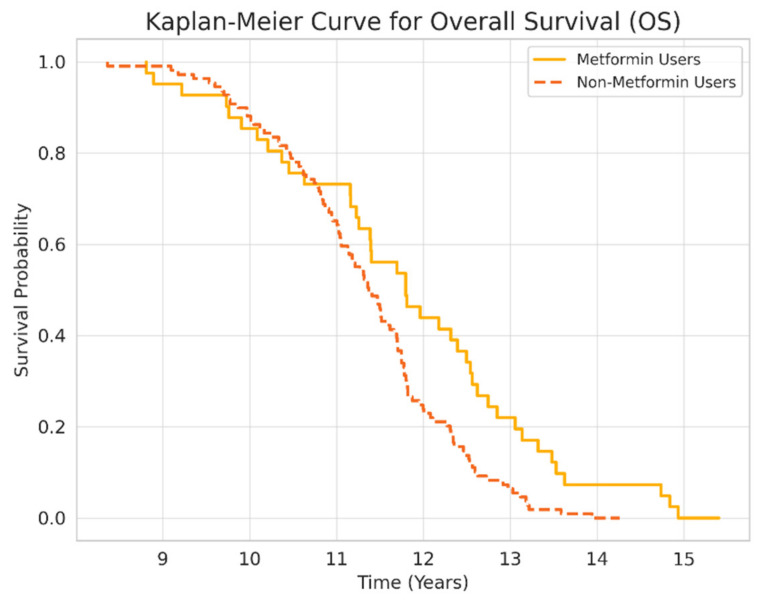
Kaplan–Meier Curve for Overall Survival (OS).

**Figure 2:**
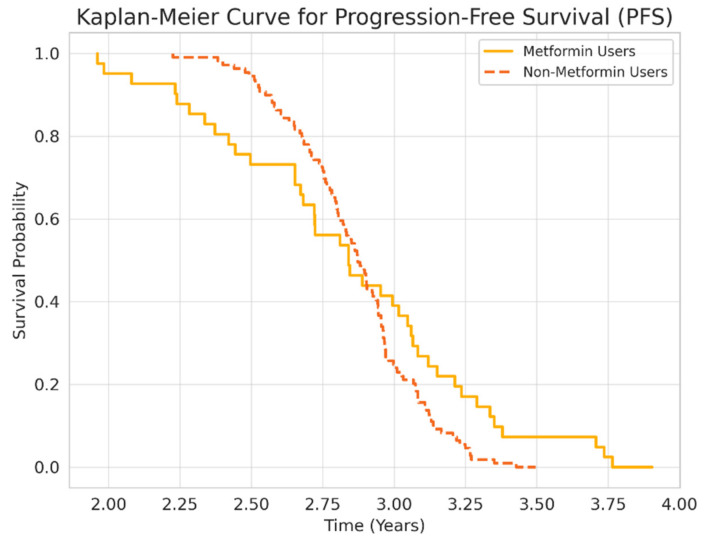
PFS Kaplan–Meier curve.

Overall survival (OS) was significantly longer in metformin users compared to non-users (5.02 ± 3.93 vs. 4.6 ± 3.79 years, *p* < 0.05). However, progression-free survival (PFS) did not differ significantly (1.32 ± 0.97 vs. 1.04 ± 0.75 years, *p* = 0.385). Kaplan–Meier survival curves (Figure 1 and Figure 2) illustrate these differences.

Table 2 summarises the Logistic Regression Analysis for Mortality Predictors. The Multivariate logistic regression analysis demonstrated that the number of nivolumab treatment cycles (median 7) was significantly associated with reduced mortality risk (OR = 0.64, 95% CI: 0.54–0.75, *p* < 0.001). Metformin use was not significantly associated with mortality reduction (OR = 0.62, 95% CI: 0.27–1.39, *p* = 0.244). Tumor histology also influenced mortality, with squamous cell carcinoma associated with increased risk (OR = 2.26, *p* = 0.044).

### 3.3. Treatment-Related Toxicities

Grade 3–4 thrombocytopenia occurred in 10/42 (23.8%) metformin users compared to 8/110 (7.3%) non-users, showing a statistically significant difference (*p* < 0.05). Other adverse events, such as anemia, neutropenia, mucositis, nephrotoxicity, and hepatic toxicity, did not differ significantly between groups (all *p* > 0.05). Immune-related toxicities (e.g., pneumonitis) were also not significantly different. Adverse events were graded according to the Common Terminology Criteria for Adverse Events (CTCAE) version 5.0. Chi-square tests were used to compare the incidence of toxicities between groups. Dose reductions were defined as a decrease in nivolumab dosage, and treatment discontinuation due to toxicity occurred in fewer than 5% of patients. Thrombocytopenia was managed with dose adjustments or platelet transfusions when clinically indicated. Table 3 summarizes the incidence of grade 3–4 adverse events in each group.

This table summarizes the number and percentage of patients experiencing grade 3–4 adverse events in the metformin and non-user groups. Adverse events were graded according to CTCAE v5.0.

**Figure 3:**
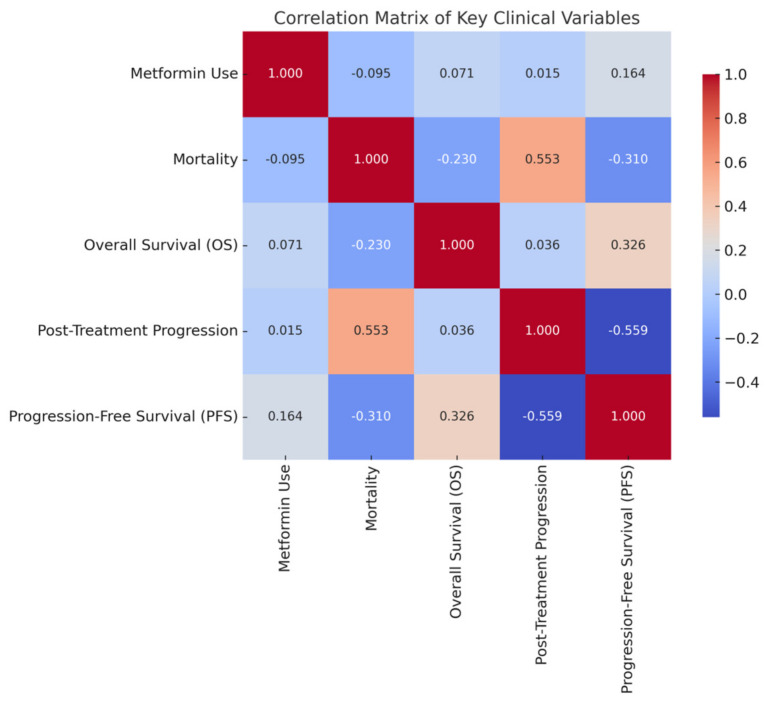
Heatmap of Spearman correlations.

Figure 3 also summarises Heatmap of Spearman correlations.

### 3.4. Correlations and Clinical Variables

Spearman’s rho correlations are reported in Table 4. Spearman correlation analysis showed r = 0.180, *p* = 0.026 for metformin use and BMI association between metformin use and higher BMI (r = 0.180, *p* < 0.05) as well as gender (r = −0.314, *p* < 0.01), reflecting the imbalance in baseline characteristics. Metformin use was not significantly correlated with OS or mortality. The weak correlation between metformin use and BMI suggests limited clinical impact. A r = 0.405, *p* < 0.001 for nivolumab cycles and OS was observed between the number of nivolumab cycles and OS (r = 0.405, *p* < 0.05), and a negative correlation between cycle number and mortality (r = −0.587, *p* < 0.05). No multicollinearity was detected among correlated variables. These findings highlight treatment duration as a key determinant of survival. Table 4 summarizes Correlation Matrix for Metformin Use, Mortality, Survival, and Other Variables.

Table 5 also summarizes the Baseline Characteristics After Propensity Score Matching (1:1).

## 4. Discussion

### 4.1. Interpretation of Key Findings

This study demonstrates that concurrent metformin use was associated with significantly improved overall survival (OS) among patients with metastatic NSCLC treated with nivolumab. However, progression-free survival (PFS) did not show a significant difference between groups. These findings suggest that metformin may contribute to enhanced T-cell responses or reduced MDSC activity rather than short-term tumor control.

### 4.2. Clinical Implications

The observed survival benefit associated with metformin use supports its potential role as an adjunct to immune checkpoint inhibition in NSCLC. Importantly, the increased incidence of grade 3–4 thrombocytopenia in the metformin group suggests a need for hematologic monitoring in patients undergoing this combination [10]. These findings are particularly relevant in light of metformin’s accessibility and low cost. This survival benefit may be attributed to immunometabolic effects of metformin, such as activation of AMPK and reduction in MDSCs, which can enhance T-cell-mediated anti-tumor responses [3,4].

### 4.3. Comparison with Existing Studies]

Our observed overall survival benefit (5.02 ± 3.93 vs. 4.6 ± 3.79 years, *p* < 0.05) contrasts with previous studies that reported minimal or no benefit of metformin in the context of immunotherapy. Buti et al. found no OS advantage in a cohort of various cancer types, which may have masked metformin’s effect in NSCLC-specific settings. The inclusion of mixed cancer types may have diluted any potential metformin effect.

Reference [11] similarly reported variable outcomes, attributing the lack of consistency variability in PD-L1 expression or tumor microenvironment. Our sample size of 42 metformin users improves statistical power and enables more robust interpretation, particularly within an NSCLC-focused cohort [8], who limited their analysis to NSCLC, reported improved progression-free survival (PFS) with metformin, although our study did not replicate this effect (PFS, *p* = 0.385).

Study design differences further explain divergent results. While Buti’s and Shen’s analyses were retrospective, Chiang’s larger NSCLC cohort offered greater statistical power and identified subgroup-specific effects [12]. Our study, though retrospective, demonstrated a significant difference in hematologic toxicity (grade 3–4 thrombocytopenia, *p* < 0.05), a finding not reported in Buti or Shen. Additionally, demographic imbalances—such as the higher proportion of female patients in the metformin group [13].

Our survival gain (5.02 vs. 4.6 years, *p* less than 0.05) contrasts with studies finding no metformin advantage in immunotherapy. In advanced cancer patients on immune checkpoint inhibitors, metformin users saw a small but statistically insignificant rise in survival lost in the accompanying broad cancer type noise, said) [14], and found no apparent survival rise. Reference [11] explored this, observing that its effects varied: some groups did well, but others did not, and linked the reason to tumor biology or limitations to a sample size of metformin use. Although modest in power (99.2%, G*Power), our 42 metformin users top their numbers (28 in Buti), boosting our power (and theirs, too) thanks to an n of 1. Cancer type plays a role, too. Reference [8] opted for lung cancer and thus exploited progression-free survival gains that we did not capture (*p* = 0.385).

Study design differences shed light. Setting up our and Buti’s perspectives and Sten’s retrospective as they grapple with real-world factors (diabetes, dosing, and more) not present in controlled trials. Smoothing these issues, Chiang’s larger cohort (though our 152 patients felt it) could spot trends we might miss. Shen and Buti missed any toxicity spikes; we are different in that we have thrombocytopenia [*p* less than 0.05] for no reason other than to say we are different [15]. It is no accident that we used gender and BMI imbalances influenced survival outcomes; others did not; we also had a gender tilt (33.3% vs. 8.2% women, *p* less than 0.05). However, the gaps in sample size, cancer mix, and focus make our survival and toxicity findings sharper, but narrower and prospective, well-powered studies are required to resolve this debate.

### 4.4. Limitations

This study has several limitations. As a retrospective analysis, it is subject to biases inherent in observational data and lacks the rigor of randomized control [2]. Our relatively small metformin group (n = 42) compared to the non-user group (n = 110) may limit generalizability, despite an estimated power of 99.2%. Furthermore, progression status data were missing in 23.8% of metformin users and 41.8% of non-users, reducing the reliability of PFS comparisons (*p* = 0.385).

Confounding factors were not fully adjusted for. In particular, diabetes severity and glycemic control (e.g., HbA1c levels) were not analyzed, limiting interpretation of survival differences. Some non-users may have had diabetes but were not receiving metformin, introducing group overlap. Moreover, significant baseline imbalances in gender (33.3% vs. 8.2%) and BMI (26.53 vs. 24.97) may reflect diabetes-related characteristics rather than direct metformin effects. Metformin may enhance nivolumab efficacy via AMPK-mediated immune activation.

While this study initially included a broader cancer cohort, we restricted our analysis to NSCLC subtypes (e.g., adenocarcinoma, squamous cell carcinoma) to ensure disease-specific relevance. The observed increase in thrombocytopenia among metformin users (*p* < 0.05) lacks a mechanistic explanation; the underlying platelet dynamics remain speculative.

Taken together, these limitations suggest the need for prospective studies with balanced cohorts, clearly defined diabetes status, and mechanistic endpoints. Future trials should evaluate metformin’s impact in well-matched NSCLC populations receiving immune checkpoint inhibitors like nivolumab.

Our study carries flaws. Retrospective design utilizes records and not controlled settings; thus, it is hard to pin cause to effect. Our metformin group (n = 42) is small compared to nonusers (n = 110), so with 152 patients, we trim statistical strength even with 99.2% power. Of course, data stings too, and progression status is missing out for 23.8 per cent of metformin users and 41.8 per cent of nonusers; progression-free survival reads (*p* = 0.385) are weakened. It closes down this hole that narrows our view on a frontier for pace, a dice roll in our view of disease pace.

Con-founders add murk. We did not adjust for how much or how tightly diabetes fuels metformin use, which might sway survival or toxicity; however, nonusers include some people with diabetes whose drug covers their diabetes, but not all other drug interventions, and the two groups are blurred. Matching differences in gender (33.3% vs. 8.2% women, *p* less than 0.05) and body mass index (26.53 vs. 24.97) could relate more closely with diabetes traits than metformin effects. Noise is brought in by cancer type variety (lung, renal, salivary), or noise is removed if the signal is evident in a broader regime. Uptick thrombocytopenia (*p* less than 0.05) does not have definite support; platelet mechanics stands for guessed but not proven. Without these, they restrict our claims and send future research toward prospective designs, larger metformin groups, and matched controls for diabetes to really zero in on the role of metformin in the fight between nivolumab and nivolumab. While propensity score matching was employed to minimize confounding, the matching process did not include laboratory markers such as lymphocyte or monocyte counts. This may have contributed to residual differences between groups. Additionally, the inclusion of mixed histologic subtypes, although balanced post-matching, may not fully account for biological variability in treatment response.

## 5. Conclusions

This study suggests that concurrent metformin use may confer a modest overall survival benefit in patients with metastatic non-small cell lung cancer (NSCLC) treated with nivolumab, without significantly affecting progression-free survival. However, metformin use was also associated with an increased incidence of grade 3–4 thrombocytopenia, indicating a need for closer hematologic monitoring. The survival advantage observed may reflect long-term immunometabolic modulation rather than early disease control.

Despite using propensity score matching to balance baseline characteristics, residual imbalances—particularly in gender and histologic subtypes—highlight the limitations of this retrospective analysis. These findings underscore the need for prospective, controlled studies to validate metformin’s role as an adjunct to immune checkpoint inhibition in NSCLC.

## Figures and Tables

**Table 1:** Demographic and laboratory comparisons.

Variable	Metformin Users (n = 42)	Non-Users (n = 110)	*p*-Value	Significance
Gender				Significant
Women (%)	14 (33.3%)	9 (8.2%)	<0.05	Yes
Men (%)	28 (66.7%)	101 (91.8%)		
Age ≥ 65 years (%)	24 (57.1%)	42 (38.2%)	0.054	No
Body Mass Index (BMI)				Not Significant
Mean (SD)	26.53 (4.54)	24.97 (4.72)	0.065	No
Normal (%)	16 (38.1%)	53 (48.2%)	0.501	No
Obese (%)	7 (16.7%)	14 (12.7%)		
Height (cm, Mean ± SD)	165.9 (7.84)	169.55 (6.65)	<0.05	Yes
Laboratory Markers				Not Significant
Lymphocytes (Mean ± SD)	1830.24 (1059.01)	1606.82 (739.23)	0.380	No
Neutrophils (Mean ± SD)	5183.1	4873.27	0.275	No
Platelets (Mean ± SD)	295,704.3	275,563.64	0.165	No
Monocytes (Mean ± SD)	604.52	785.27	0.281	No

All laboratory parameters are expressed in ×10^9^/L.

**Table 2:** Logistic Regression Analysis for Mortality Predictors.

Predictor Variable	B (Coefficient)	S.E. (Standard Error)	*p*-Value	OR (95% CI) (Odds Ratio, OR)	95% CI for OR (Lower–Upper)
Age	−0.05	0.36	0.885	0.95	0.47–1.90
Gender (Female = 0, Male = 1)	−0.43	0.47	0.357	0.65	0.26–1.63
BMI	0.01	0.04	0.831	1.01	0.94–1.08
Histopathology (ADC = Ref.)					
Squamous cell carcinoma (SDC)	0.82	0.41	0.044	2.26	1.02–5.01
Mixed Type	0.91	0.50	0.069	2.49	0.93–6.65
Other Types	2.01	1.26	0.110	7.47	0.63–88.02
Nivolumab Cycles	−0.45	0.08	0.000	0.64	0.54–0.75
Metformin Use (Yes = 1, No = 0)	−0.48	0.42	0.244	0.62	0.27–1.39

ADC: Adenocarcinoma, used as reference category. Confidence intervals were calculated using the Wald method. Nagelkerke R^2^ for the model was 0.35.

**Table 3:** Incidence of Grade 3–4 Adverse Events by Group.

Adverse Event	Metformin Users (n = 42)	Non-Users (n = 110)	*p*-Value
Thrombocytopenia	10 (23.8%)	8 (7.3%)	<0.05
Anemia	5 (11.9%)	12 (10.9%)	>0.05
Neutropenia	3 (7.1%)	9 (8.2%)	>0.05
Mucositis	2 (4.8%)	4 (3.6%)	>0.05
Nephrotoxicity	1 (2.4%)	3 (2.7%)	>0.05
Hepatic toxicity	2 (4.8%)	3 (2.7%)	>0.05
Pneumonitis (immune-related)	1 (2.4%)	2 (1.8%)	>0.05

**Table 4:** Correlation Matrix for Metformin Use, Mortality, Survival, and Other Variables.

Variables	Metformin Use	Mortality Risk	Overall Survival (OS)	Disease Progression	Progression-Free Survival (PFS)
Metformin Use	1.000	−0.095	0.071	0.015	0.164
Mortality Risk	−0.095	1.000	−0.230	0.553	−0.310
Overall Survival (OS) (years)	0.071	−0.230	1.000	0.036	0.326
Disease progression Status	0.015	0.553	0.036	1.000	−0.559
Progression-Free Survival (PFS) (years)	0.164	−0.310	0.326	−0.559	1.000

**Table 5:** Baseline Characteristics After Propensity Score Matching (1:1).

Variable	Metformin Users (n = 42)	Matched Non-Users (n = 42)	*p*-Value	SMD
Age (mean ± SD)	65.1 ± 7.3	64.8 ± 6.9	0.81	0.04
Female (%)	33.3%	31.0%	0.82	0.05
BMI (mean ± SD)	26.5 ± 4.5	26.3 ± 4.4	0.74	0.03
Histology (ADC %)	76.2%	78.6%	0.79	0.06
Nivolumab Cycles (mean)	7.1 ± 2.3	7.0 ± 2.5	0.88	0.02

Note: ADC = Adenocarcinoma; SMD = Standardized Mean Difference.

## Data Availability

No new data were created or analyzed in this study. Data sharing is not applicable to this article.
